# Extensive Cellulitic Infection and Tissue Necrosis in a Patient With Hyper IgE Syndrome: Surgical Management of a Rare Immunodeficiency

**DOI:** 10.7759/cureus.89247

**Published:** 2025-08-02

**Authors:** Quinton L Carr, Colton H Connor, Ryan A Cantrell, Shriya D Dodwani, Alyssa Nguyen, Joshua D Spiegel, Brian J Paul, Joshua H Choo

**Affiliations:** 1 Plastic and Reconstructive Surgery, University of Louisville School of Medicine, Louisville, USA; 2 Plastic and Reconstructive Surgery, University of Louisville Hospital, Louisville, USA

**Keywords:** hyper ige syndrome, hyper immunoglobulin e syndrome, job’s syndrome, split-thickness skin graft, two-stage reconstruction

## Abstract

Hyper IgE syndrome (HIES) is a rare primary immunodeficiency that is characterized by elevated serum IgE levels, recurrent sinopulmonary infections, and chronic eczema, among other symptoms. Though reports on patients with HIES exist, they primarily focus on the clinical features, diagnosis, and management of HIES without detailing surgical interventions.

Here, we present the surgical management of an extensive cellulitic infection that developed into skin necrosis in an HIES patient with a history of polysubstance abuse. The patient had a diagnosis of Job’s syndrome, the autosomal dominant form of HIES. In this case, severe infection resulting from immunodeficiency led to cellulitis and full-thickness tissue loss in the left upper limb. Due to the extent of infection and necrosis, multiple debridements and upper extremity reconstructions were necessary for limb preservation.

The 625 cm² wound, which involved the left upper extremity and crossed the elbow joint, was managed with initial debridement followed by a period of local wound care to allow for clearance of infection. We then performed reconstruction in a staged fashion with dermal substitute (Integra, Integra LifeSciences Holdings Corporation, Princeton, NJ) placement and split-thickness skin grafting (STSG). In this case, we demonstrate that the extensive soft tissue infections that may be found in HIES patients can be successfully managed with skin grafting and dermal substitutes.

## Introduction

Hyper IgE syndrome (HIES) is a rare primary immunodeficiency disorder with only around 300 cases reported in the literature [1-3]. HIES is characterized by significantly elevated serum IgE levels, chronic eczema, and increased susceptibility to soft tissue infections, particularly of the skin and lungs [1,4,5]. The majority of HIES cases are sporadic, though familial inheritance has also been observed [5].

The two primary categories of HIES are distinguished by either autosomal dominant or autosomal recessive inheritance [1,6]. Autosomal dominant HIES (AD-HIES), first termed “Job's syndrome,” is the result of a STAT3 deficiency and is the cause of HIES in roughly 70% of cases [1,5]. STAT3 is broadly involved in the immune response and healing, as the STAT3 protein serves as a transcription factor in the signaling pathways of various pro- and anti-inflammatory cytokines, including IL-6, IL-10, IL-11, and IL-21 [6]. IL-6 is especially important in the immune response, as it acts as the third signal needed for CD4 T cell differentiation to the Th17 helper T cell subtype [6]. Th17 cells are essential for combatting bacterial and fungal infections [1,6], and reduction of this cell subtype leads to underproduction of IL-17, a chemotactic agent used to recruit monocytes and neutrophils to the site of inflammation. IL-10 is an anti-inflammatory cytokine and can lead to the formation of immunosuppressed microenvironments by regulating the production of pro-inflammatory cytokines such as IL-1, IL-6, and TNF-alpha [6]. The reduced immunity from the disruption of IL-6 and IL-10 seen in HIES patients often leads to recurrent skin infections, most commonly caused by *Staphylococcus aureus* (*S. aureus*) and *Candida*. Such infections can often present as cold staphylococcal skin abscesses. Pulmonary infections, such as recurrent pneumonia, are also common and due primarily to *S. aureus*, although they are sometimes caused by *Streptococcus pneumoniae* (*S. pneumoniae*) or *Haemophilus influenzae* (*H. influenzae*) [1]. IL-11 plays a role in the ossification of cranial sutures, primary teeth exfoliation, and other osteological functions; thus, disruption of IL-11 signaling contributes to the hallmark skeletal abnormalities seen in AD-HIES. These include coarse facial features, delayed primary tooth shedding, and susceptibility to bone fractures, among others [5,6]. IL-21 is involved in the differentiation of B-cells into plasma cells. Typically, anti-inflammatory contributions from IL-10 and IL-21 contribute to the suppression of IgE production by B cells. However, impaired signaling of these cytokines leads to the high serum IgE levels seen in AD-HIES [6]. The disruption of these signaling pathways by STAT3 deficiency contributes to the manifestation of AD-HIES symptoms.

Autosomal recessive HIES (AR-HIES) is a condition usually caused by mutations in the DOCK8 gene. However, mutations in PGM3, CARD11, and ZNF431 have also been observed in association with the HIES phenotype [6]. Additionally, several distinct primary immunodeficiency disorders have been identified that mimic many symptoms of HIES, making it difficult to distinguish between these disorders. However, the treatment options differ between conditions, so this distinction is important [6].

Currently, there are no definitive cures for HIES. Patients are currently managed prophylactically with antimicrobials and meticulous skin care [6]. Reports of success with intravenous immunoglobulin (IVIG) exist, though this application is controversial, and conclusions regarding IVIG efficacy in HIES patients cannot be confidently drawn at this time [5]. Recently, success has been observed following hematopoietic stem cell transplantation in both AD-HIES and AR-HIES patients, despite failure in earlier trials [7]. Further advancements in gene therapy may provide for more effective treatments in the future [1].

In this report, we present the surgical resolution of cellulitis that developed into skin necrosis in a patient who was diagnosed with AD-HIES by the NIH. Multiple operations were required due to the extent of the patient’s infection. Preservation of the left upper limb was achieved following serial excisional debridement, placement of a dermal regeneration template (Integra, Integra LifeSciences Holdings Corporation, Princeton, NJ), and skin grafting. To our knowledge, there has been only one previously reported case of an HIES-related infection treated with skin grafting [8], and our patient is the first to receive a dermal regeneration template prior to graft placement.

## Case presentation

**Figure 1:**
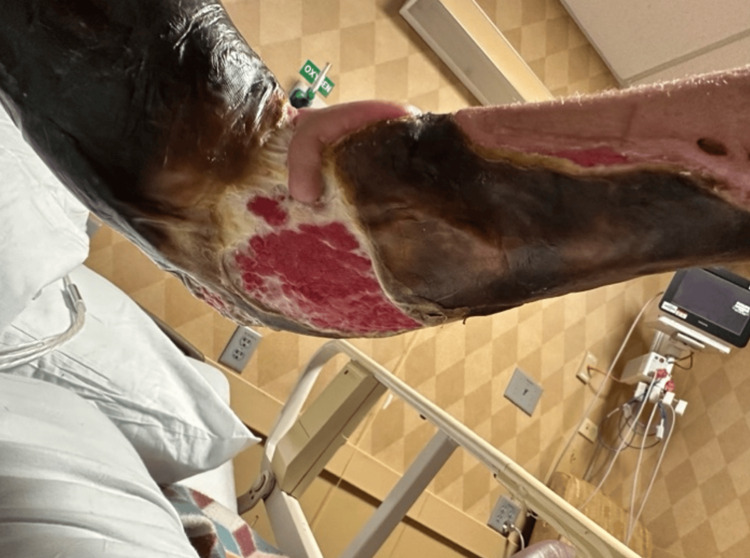
First visual of the wound at the time of initial consultation by the University of Louisville plastic surgery team.

**Figure 2:**
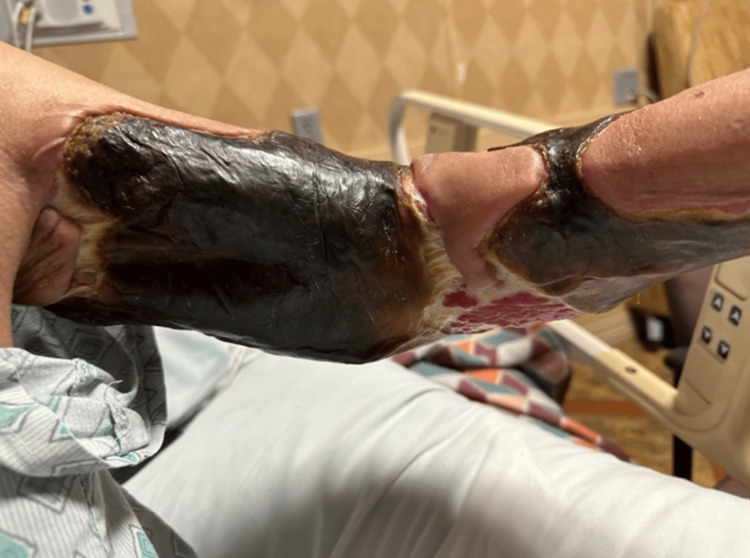
Second visual of the wound at the time of initial consultation by the University of Louisville plastic surgery team.

**Figure 3:**
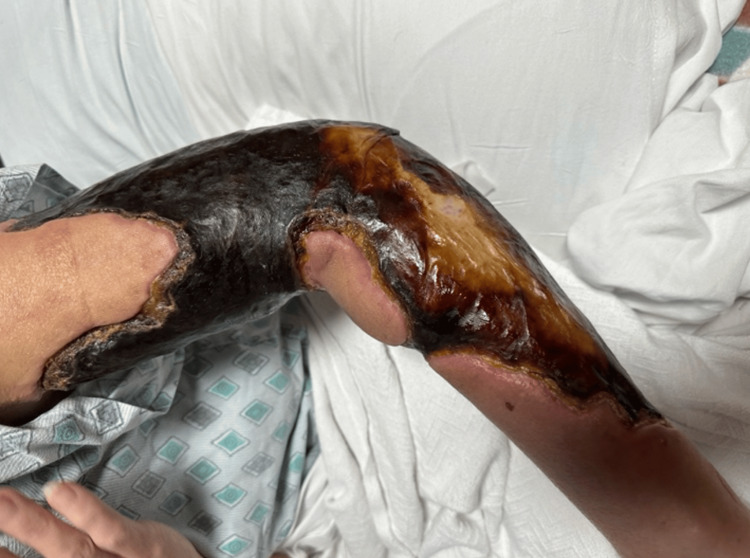
Third visual of the wound at the time of initial consultation by the University of Louisville plastic surgery team.

The patient was a 45-year-old female who first presented to the University of Louisville with extensive cellulitis of the left breast. First managed medically, the cellulitis was eradicated from the breast but spread to the left upper extremity over the course of several months. During this time, she received care from a number of local hospitals, complicating her treatment and follow-up. According to the patient, the wound began with cysts, then developed into blood blisters and cellulitis. Previously, she had suffered from several of the classic HIES symptoms, including recurrent abscesses and pneumonia. She later experienced central stenosis of the spinal canal and chronic osteomyelitis of the cervical spine. When the University of Louisville plastic surgery team was first consulted, six months after her initial presentation, the wound had progressed into extensive cellulitis and skin necrosis in her left upper extremity. Visuals of the wound at the time of initial consultation are displayed in Figures 1-3. At this time, she was seeking a second opinion after refusing the debridement recommended by another hospital.

The patient’s social history indicated that she was an everyday smoker who smoked five or more cigarettes per day. Additionally, she had a history of daily polysubstance abuse, which included heroin, marijuana, methamphetamine, and alcohol.

At the time of evaluation, the wound had developed into a circumferential eschar extending from the axilla to the mid-forearm with a skin island over the antecubital fossa. There was a bare area of granulation tissue over the medial condyle. Excisional debridement was performed, revealing purulent necrotic tissue underlying the eschar on the upper arm, though healthy hypergranulation tissue was present in the forearm. At its deepest, the wound penetrated to muscle, but there was no exposed bone. A tissue culture and stain were ordered, and the result was positive for *S. aureus*, a common offender in both HIES patients and IV drug users. We proceeded with local wound care of wet-to-dry dressings to allow for continued mechanical debridement and for granulation tissue to form prior to skin grafting.

**Figure 4:**
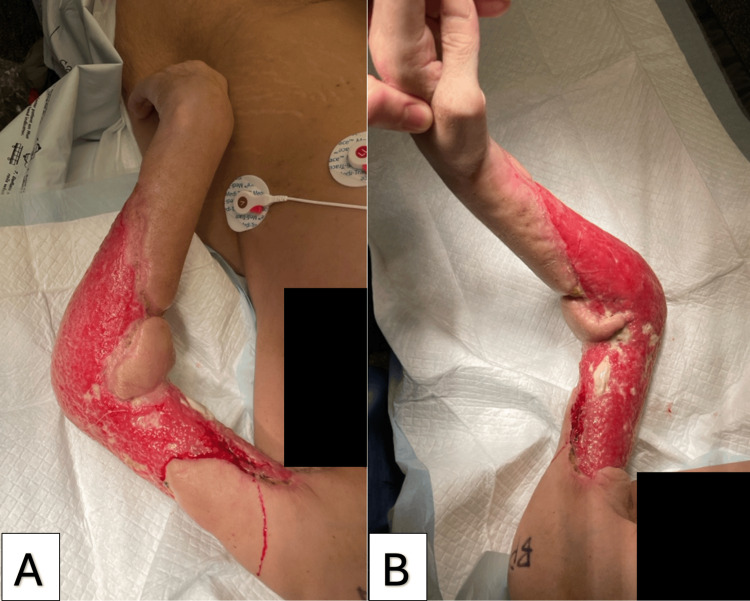
Visual of the wound after nearly one month of local wound care and clearance of infection.

Nearly one month later, after clearance of any infection, the patient was deemed ready for grafting. Visuals of the wound at this stage are shown in Figure 4. Due to the vast extent of the wound, it was decided that, at first, only a portion of the wound would receive a skin graft. A 0.014” split-thickness skin graft (STSG) was harvested from the left thigh using a dermatome and meshed at a 1.5:1 ratio. The graft was then placed on the forearm, where the wound appeared the healthiest, covering an area of approximately 125 cm². After securing the graft with staples, the remaining 500 cm² of the wound was covered with Integra (Integra® Dermal Regeneration Template (IDRT)). The graft was dressed with Adaptic (3M™ Adaptic™ Non-Adhering Dressing, Solventum Corporation, Maplewood, MI), and the entire wound was wrapped in Acticoat (ACTICOAT Surgical Dressings, Smith & Nephew PLC, Watford, United Kingdom). Lastly, a wound vacuum-assisted closure (VAC) (3M™ V.A.C.® Therapy, Solventum Corporation) was applied as a bolster.

**Figure 5:**
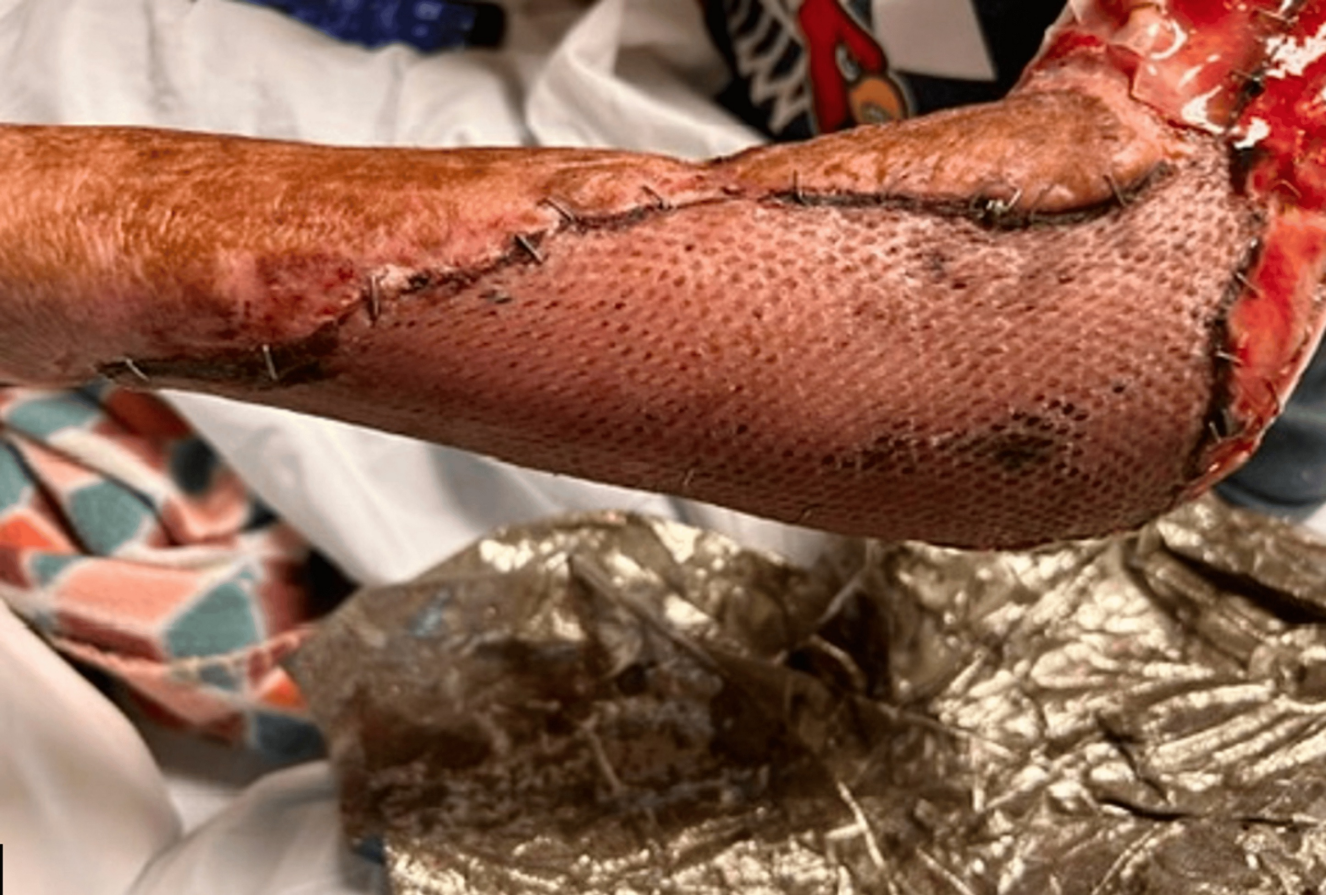
Image of the initial skin graft after a period of 25 days.

**Figure 6:**
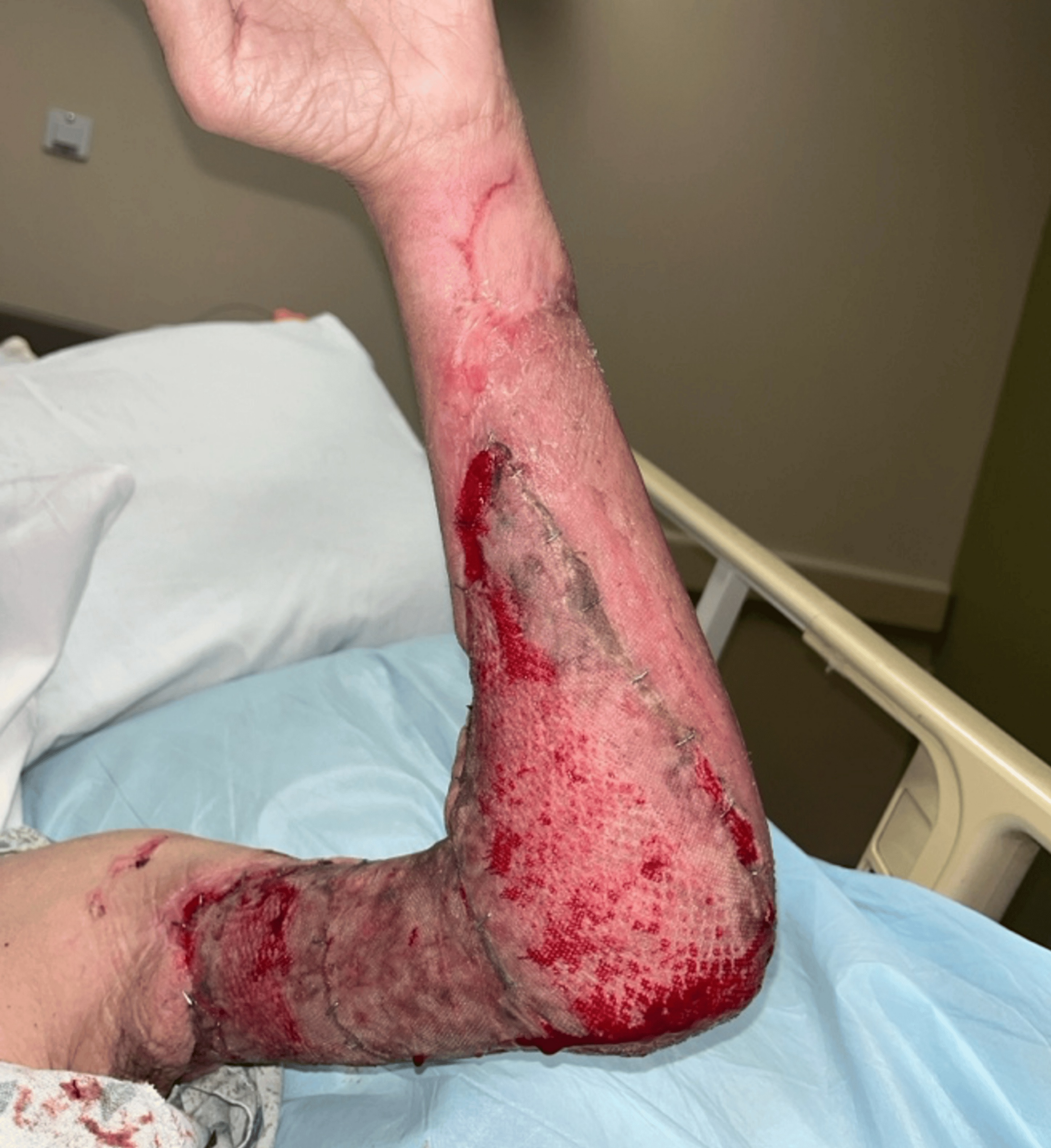
Second-stage graft on postoperative day 5, showing successful graft take.

Figure 5 illustrates the progress of the initial graft after 25 days. At that time, the neodermis covering the remaining wound had vascularized, and the patient was ready for second-stage skin grafting [9]. An area of 366 cm² was prepared, and a 0.014” STSG was harvested from the left thigh using a dermatome and applied as a sheet to the antecubital fossa. Another 0.014” STSG was harvested from the right thigh, meshed in a 1.5:1 pattern, and applied to the remaining areas. These grafts were secured with staples, pie crusting incisions, and mattress sutures. The arm was then covered with Acticoat, and a wound VAC. Figure 6 depicts the second-stage graft on postoperative day five, demonstrating a satisfactory graft take. The patient tolerated the procedures well with no intraoperative or postoperative complications and was discharged to a homeless care facility after 19 days.

## Discussion

HIES is a rare primary immunodeficiency disorder characterized by elevated levels of IgE and a broad alteration of inflammatory response [1,4,5]. Reduction of Th17 cells leads to underproduction of IL-17, a chemotactic agent used to recruit monocytes and neutrophils to the site of inflammation. Low levels of IL-17, as well as a myriad of immune function losses associated with a STAT3 deficiency, manifest as “cold” soft tissue abscesses, recurrent pneumonias, chronic eczema, connective tissue abnormalities, skeletal defects, and an increased susceptibility to skin and lung infections, the classic symptoms of AD-HIES. The reduced immunity in HIES can most commonly predispose the patient to infections by S. aureus and Candida [1]. S. aureus is also the hallmark bacterial pathogen in infections among intravenous drug users (IVDU), and methamphetamine has been shown to exacerbate S. aureus skin and soft tissue skin infections (SSTI), leading to a compounding risk of infection in IVDUs who are also immunocompromised, such as our patient [10]. A summary of HIES can be found in Table 1. 

**Table 1:** Hyper IgE syndrome summary Sources: [1,6]

Hyper-IgE syndrome	
Clinical manifestations	Recurrent pneumonia, chronic eczema, “cold” skin abscesses, connective tissue, and skeletal abnormalities
Diagnostic criteria	Genetic testing for STAT3 mutations (autosomal dominant), DOCK8 mutations (autosomal recessive), elevated serum IgE, and eosinophilia
Common therapeutic methods	Prophylactic antimicrobials (antibiotics, antifungals) and meticulous skin care
Prognosis	Historically poor morbidity and mortality outcomes; significantly improved with adequate prophylactic and supportive care.

Due to the large extent of the patient’s wound after excisional debridement, an STSG was deemed the most appropriate method for reconstruction, given the size of the wound. Full-thickness skin grafts (FTSG) are contraindicated for large, avascular wounds greater than one centimeter, as they require better perfusion than STSGs and produce a more severe wound at the donor site [11]. Due to their reduced thickness, STSGs require less vascular supply than FTSGs, often making them a better option in smokers, as smoking impairs wound healing and oxygenation [12, 13].

In this case, grafting was performed in two stages, given the lack of granulation tissue in areas throughout the wound. In stage 1, the first STSG was meshed, as meshing increases the size of skin grafts while maintaining a minimal donor site defect [14]. The graft was then placed on the forearm, where sufficient granulation tissue was located.

After the placement of the first STSG, an IDRT was placed on the remaining area to help prepare a bed for subsequent grafting. IDRT promotes dermal regeneration through the use of a synthetic, porous bilayer membrane consisting of a type 1 collagen-glycosaminoglycan layer and a semi-permeable silicone cover. Its structure and bioengineered composition promote fibroblast migration, neovascularization, and tissue remodeling, which optimize neodermal formation and wound healing [9, 15]. Use of an IDRT and other bilayer matrices is often indicated for the management of partial and full-thickness wounds, surgical wounds, trauma and burn wounds, and draining wounds, among others [9]. After sufficient time has passed to allow for the proliferation of fibroblasts and neovascularization of new tissue, the color of the silicone layer is assessed for a pink/peach coloration, which indicates adequate healing [9, 16]. The superficial silicone layer is subsequently removed, and a skin graft is placed [9, 17]. For this case, the IDRT was removed after 25 days, following the timeline of neodermis revascularization noted in the literature [18].

After the IDRT was removed, we could proceed with the second stage of grafting and ultimately close the wound. In this stage, the second and third grafts were placed. The second was applied unmeshed due to the small size of the wound in the antecubital fossa, and the third was meshed and applied to the remaining areas.

Patients with HIES are known to exhibit poor wound healing [19], which further compounds the infection risk associated with the immunodeficiency. Therefore, we employed various advanced wound care products to aid in infection control and promote healing. The STSGs and the donor sites were treated with Adaptic, a cellulose acetate mesh coated with a petrolatum emulsion that promotes wound healing by promoting epithelialization, reducing infection risk, and absorbing exudates [20].

Acticoat was also applied as a protective covering at both the initial graft placement and after grafting onto the vascularized neodermis 25 days later. Animal trials with Acticoat suggest that the slow release of silver-impregnated nanocrystals into the wound exudate has a broad spectrum of antibiotic activity and is effective against a host of common wound pathogens [21]. Human clinical trials, though few in number, have suggested that Acticoat offers an economical solution for wound management by decreasing matrix metalloproteinase activity, lowering the risk of infection, reducing the need for frequent dressing changes, minimizing wound exudate and microbial load, and aiding in the healing of chronic wounds [21].

Wound VAC was applied as a final means to facilitate healing. The existing literature indicates that gentle negative pressure wound therapy applied to the site of defect can reduce swelling through the removal of excess fluids and exudates, thereby also decreasing bacterial bioburden and lowering the risk of infection [22]. Proper debridement prior to VAC use can further reduce the risk of infection [23, 24]. Additionally, VACs have been shown to promote oxygen and nutrient delivery to the wound by increasing blood flow [22]. The pro-healing attributes of VACs also support the formation of granulation tissue, which is essential for wound closure [22]. VACs have been specifically indicated for use in skin graft healing, with studies indicating that VACs can increase rates of skin graft take and survival compared to standard bolster dressings and may also produce a better cosmetic result [23, 25, 26].

Given the wound characteristics and method of reconstruction used in this case, contracture was a primary concern. Despite the benefits of STSGs, they are more prone to contracture, and the effects of contracture may be compounded by joint-crossing grafts, as joint contracture may severely limit mobility [13, 27]. Ideally, physical therapy would be employed to help maximize range of motion and reduce joint stiffness resulting from inactivity and scar formation [28]. Unfortunately, the patient has not attended long-term follow-up, so long-term complications have not been evaluated, and she did not receive the care needed to minimize these complications.

Despite her primary immunodeficiency, our patient successfully healed from extensive cellulitic infection and soft tissue necrosis with the support of advanced wound healing technologies, including Integra, Adaptic, Acticoat, and VAC. To our knowledge, the patient did not experience any intraoperative or postoperative complications.

## Conclusions

This case highlights a successful two-stage wound reconstruction in a patient with HIES. To our knowledge, this is the first reported instance where advanced wound healing technologies, such as dermal substitutes, Adaptic, Acticoat, and wound vacuums, have been utilized in wound reconstruction in a patient with HIES and is the second reported instance of skin grafting. This report suggests that positive surgical outcomes in patients with HIES are possible despite the condition being associated with poor wound healing and a high risk of infection. Future reports detailing surgical intervention in this demographic may assist in determining best practices.
